# Spray-drying Microencapsulation of an Extract from *Tilia tomentosa* Moench Flowers: Physicochemical Characterization and *in Vitro* Intestinal Activity

**DOI:** 10.1007/s11130-022-00995-y

**Published:** 2022-08-10

**Authors:** Federica Mainente, Anna Piovan, Francesca Zanoni, Roberto Chignola, Silvia Cerantola, Sofia Faggin, Maria Cecilia Giron, Raffaella Filippini, Roberta Seraglia, Gianni Zoccatelli

**Affiliations:** 1 Department of Biotechnology, University of Verona, Strada Le Grazie, 15 - CV1, 37134 Verona, Italy; 2 Department of Pharmaceutical and Pharmacological Sciences, University of Padova, Via Marzolo, 5 - 35131 Padova, Italy; 3 Sphera Encapsulation SRL, Via Alessandro Volta, 15A - 37062 Villafranca di Verona, Verona, Italy; 4 IRCCS San Camillo Hospital, Via Alberoni, 70 - 30126 Venice, Italy; 5 CNR-ICMATE, Corso Stati Uniti, 4 - 35127 Padova, Italy

**Keywords:** Silver linden (*Tilia tomentosa* Moench), Encapsulation, Flavonols, Phenolic compounds, Octenyl succinic anhydride-modified starch, Intestinal contractile response

## Abstract

Silver linden (*Tilia tomentosa* Moench, *TtM*) flowers possess several health-promoting properties, especially at the neurological level, such as intestinal relaxation activity associated with specific flavonols, particularly quercetin and kaempferol derivatives. However, such molecules are susceptible to degradation upon different triggers like heat, light and extreme pH values. To overcome the scarce stability of *TtM* flowers bioactive molecules and make them suitable for developing functional food and supplements, we applied microencapsulation. Spray-drying microencapsulation of *TtM* flowers extract was performed using three starch-derived wall materials: maltodextrin 12 DE (MD12) and 19 DE (MD19), and OSA-modified starch (OSA-S). The stability of total phenols, flavanols, and antioxidant capacity was monitored for 70 days under accelerated stress conditions (40 °C/70% RH) by HPLC and spectrophotometric methods, and the intestinal contractile activity was tested in a murine model. In comparison to MD12 and MD19, OSA-S stood out for the higher encapsulation efficiency of quercetin and kaempferol glycosides (+ 36–47% compared to MD12 and + 18–24% compared to MD19) and stability thereof (half-life on average + 30% compared to MD12 and + 51% compared to MD19). The intestinal contractile activity of OAS-S powders resulted comparable to the original extract, indicating that flavonols were biologically active and accessible. Our results underly the potential advantages of OSA-S encapsulated formulation as a functional ingredient for the development of nutraceutical products.

**Supplementary Information:**

The online version contains supplementary material available at 10.1007/s11130-022-00995-y.

## Introduction

*Tilia tomentosa* Moench (*TtM*), also known as silver linden, is a deciduous tree belonging to the *Malvaceae* family [1]. *TtM* dried inflorescences are traditionally used as a popular remedy against cough, sore throat (tonsillitis and/or pharyngitis) [2], cold, and bronchitis [3]. *Tilia* flowers are also used as diaphoretic, diuretic, and antispasmodic agents [4]. In recent years, numerous studies confirmed the anti-inflammatory [3], hepatoprotective [5], antinociceptive [6], anxiolytic [7], and antispasmodic properties of *Tilia* extracts [8]. These properties are frequently associated with the presence of specific flavonols, *i.e*., phenolic compounds belonging to the broader group of flavonoids acknowledged for their numerous biological properties [9]. In particular, the most active flavonols recognized in *TtM* flowers are glycosides of quercetin and kaempferol, like tiliroside, isoquercitrin, rutin, and astragalin [4]. *TtM* flowers are used worldwide in the form of infusions, decoctions, or tinctures. The composition of extracts and their biological activity may depend on the extraction technique and the solvent used [3]. Recent finding also suggest that *Tilia* flower extracts can positively modulate human gut microbiota and this could contribute to the mentioned anti-inflammatory properties [9].

The chemical instability typical of phenolic compounds, the scarce solubility in aqueous solutions of some flavonoids (e.g., quercetin), and the pronounced organoleptic properties (such as taste and smell) represent issues [10] that can limit food and nutraceutical applications of *TtM* flowers. These problems could be overcome by using microencapsulation, i.e., the process of entrapping target molecules with one or more wall (or coating) materials to protect them and improve their functionalities. Spray-drying is a largely employed microencapsulation technology by which it is possible to convert liquid extracts into powders with enhanced stability, ease of manipulation and integration into different types of functional foods and supplements [9]. Existing literature on the spray-drying of *TtM* flower extracts is limited to one study [11] based on maltodextrin 13–17 DE (dextrose equivalents) as the wall material, and no data about the stability over time and the efficacy of the encapsulated bioactive molecules are available.

Recently, we described the biological activity *in vitro *of a commercial *TtM* flower extract, providing a molecular basis for the use of *TtM* for the treatment of functional gastrointestinal disorders [8]. In the present study, we aimed at evaluating the effects of spray-drying encapsulation of the same extract using different starch-derived wall materials, *i.e*., two maltodextrins with different DE, *i.e.*, MD12 and MD19, and octenyl succinic anhydride (OSA) modified starch (OSA-S), comparing their impact on the stability of *TtM* flower flavonols, and finally confirming the pharmacological properties of the microencapsulated powders (*TtMP*) on intestinal neuromuscular activity.

## Materials and Methods (Reported in the Supplementary Material #1)

### Results and Discussion

#### *TtME* Characterization

*TtME* presented a TPC of 11.22 ± 0.68 mg GAE/mL, a TFC of 0.72 ± 0.03 mg QE/mL, and AOC of 19.70 ± 0.92 mg TE/mL (see Table S1 reported in the Supplementary Material #2). Since the extract was produced using a drug/solvent ratio of 1:1, the calculated value can also be expressed *per* gram of dry weight (dw) plant material.

Similar results can be found in previous works. Demiray et al. [12] quantified the phenolic content of *TtM* flower extract using three different solvents: 70% acetone solution, 100% water, 100% methanol. The best levels were obtained with acetone, with a polyphenolic content of 18.3 mg GAE/g dw plant, while water was the less efficient extractor with a TPC of 5.4 mg GAE/g dw. The antioxidant activity was 14.7 mg ascorbic acid equivalent/g dw plant using ABTS. Marrassini et al. [13], working on *Tilia x viridis*, obtained a greater TPC extracting with ethanol and hydroalcoholic mixtures, *i.e*., 30 mg/g dw. Akyuz et al. [14], using methanol at 60 °C as an extraction solvent for *Tilia rubra* subsp. *Caucasica* flowers obtained a TPC of 17.37 mg GAE/g dw and a TFC of 0.04 mg QE/g dw. It is interesting to note that the TFC reported by the authors is more than 10 times lower in comparison to what was found in the present work, although the TPC content was 1.6 times greater. It is likely that the discrepancy lies in the different studied plant species since, for instance, no kaempferol was detected by the authors, while in our case, this flavonol was represented by more than one derivative [8].

It is well known that the main molecules associated with the biological activity of *Tilia* flower extract, and in particular with its effects on the nervous system, are mainly kaempferol and quercetin glycosides [7, 15, 16]. For this reason, the extract was analyzed by RP HPLC-DAD focusing the detection to flavonols as recently described [8]. Briefly, the analysis of the hydrolyzed extract allowed us to identify quercetin and kaempferol as the dominant aglycones (Fig. S1 reported in the Supplementary Material #2). Based on these results, the quantitative data were reported as equivalents of these two flavonols. The contents of quercetin and kaempferol were 399 µg/mL ± 2.1 and 252 µg/mL ± 1.7 , respectively as recently found by Cerantola et al. [8]. These values are in good accordance with the TFC of the extract, confirming that the protocol of Pekal et al. [17] here adopted is suitable for quantifying the flavonols present in *TtME*. Indeed, flavanols like catechins, ordinarily present in *Tilia* ssp. extracts [18] are not detected by the adopted procedure [17].

### Encapsulation of *TtME*

We applied spray-drying technology to improve the stability of *TtME* phenolic compounds to degradation/oxidation and make them easier to manipulate. Spray-drying is a relatively cheap, simple, and scalable process that allows the dehydration of extracts and slurries in combination with specific hydrophilic polymers to form powders. We compared the performance of three different polymers as wall materials, *i.e*., OSA-S, MD12, and MD19. The chromatograms of the flavonols extracted from the three *TtMPs* are presented in Fig. S2, panels b, c, and d. Although phenolic compounds underwent a high temperature during spray-drying, no distinct qualitative differences were appreciable.

Table 1 presents the quantitative data of the three formulations in terms of total and surface phenolic compounds calculated by Folin-Ciocalteu assay (*i.e*., TPC and SPC) and by HPLC (*i.e*., TQC and SQC for quercetin, and TKC and SKC for kaempferol). Even though the three *TtMPs* gave similar TPC, MD19 was characterized by higher SPC, indicating a lower capacity of this material to bind phenolic compounds. HPLC analysis of Q and K partially confirmed these results: indeed, both MDs exhibited higher surface flavonols, but MD12 was characterized by the highest SQC and SKC. OSA-S displayed the best encapsulating properties showing a higher capacity of establishing stronger interactions with flavonols. This is probably due to the octenyl succinic groups that give the starch molecule surface-active properties, enhancing the interactions with flavonols that are less water-soluble than other phenols like hydroxycinnamic and phenolic acids.

**Table 1:** Chemical characteristics of the TtMPs

	TPC (mg GAE/g)		SPC (mg GAE/g)		TQC (mg QE/g)		SQC (mg QE/g)		TKC (mg KE/g)		SKC (mg KE/g)		Aw (%)		Moisture (%)		AOC (DPPH) (mg TE/g)		EY (%)	
	mean	SE	mean	SE	mean	SE	mean	SE.	mean	SE	mean	SE	mean	SE	mean	SE	mean	SE	mean	SE
**OSA-S**	43.99a	3.79	1.49a	0.64	1.25a	0.05	0.14a	0.01	0.75a	0.02	0.03a	0.01	0.14a	0.06	6.58a	0.06	62.47a	5.45	61.8a	3.0
**MD12**	47.26a	4.26	1.96a	0.42	1.26a	0.02	0.44b	0.02	0.69b	0.03	0.24b	0.01	0.22a	0.02	7.74a	0.23	56.44a	0.96	57.9a	4.2
**MD19**	46.07a	2.89	3.06b	0.70	1.25a	0.04	0.31c	0.01	0.70ab	0.02	0.16c	0.01	0.16a	0.05	8.07a	0.63	59.52a	6.86	59.2a	7.2

*TtMPs*: *Tilia tomentosa* Moench powders; TPC: total phenolic content; SPC: surface phenolic content; TQC: total quercetin content; SQC: surface quercetin content; TKC: total kaempferol content; SKC: surface kaempferol content; Aw: water activity; AOC: antioxidant activity; EY: encapsulation yield. Values are expressed as mean ± standard error (SE) of three independent measurements. Different letters for each column indicate significant differences (*p* ≤ 0.05).

**Fig. 1:**
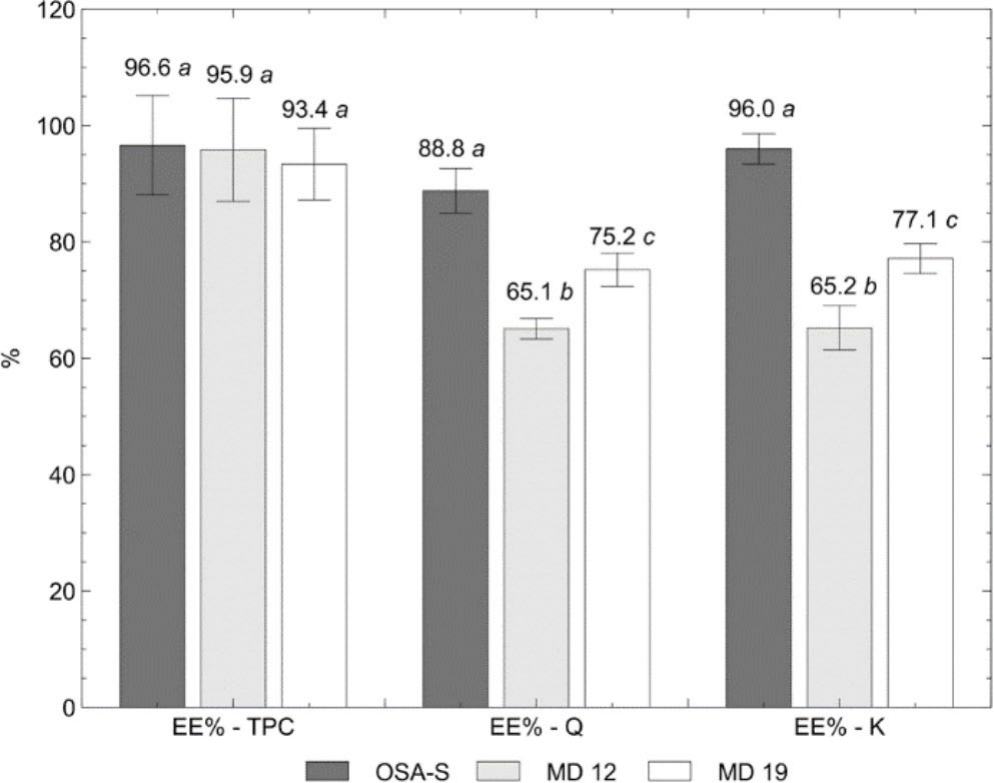
Encapsulation efficiency (EE) of total phenolic compounds (TPC), quercetin (Q), and kaempferol (K) for the three employed wall materials. Different letters for each group indicate significant differences (*p* ≤ 0.05)

These results translate into different encapsulation efficiency profiles (Fig. 1). When it comes to total phenols, no significant differences were found among the three materials, with EE ranging from 88.8 to 96.6%, not far from what Alaşalvar et al. [11] obtained using MD 13–17 DE (97.4%). On the contrary, OSA-S stood out as the best wall material to encapsulate *TtM* flavonols compared to MDs (Fig. 1). This is the first report of the encapsulation efficiency of specific *TtM* flavonoids. The fact that different maltodextrin DE performed differently is not new: previously published data highlighted the different binding capacities of MD for phenolic compounds as a function of their DE [19]. The higher amount of flavonoids on the particle surface should correlate with their faster degradation being less protected by the wall materials.

The AOC of the *TtMPs* did not show significant differences. This result was expected since the AOC was measured only for total phenols extracted from the powders, which did not exhibit differences in terms of TPC, TQC, and TKC. Concerning Aw and moisture content, no significant differences were observed between the three different *TtMPs.* Even the encapsulation yield (EY), a parameter strongly associated with the profitability of the process, did not significantly vary, showing values close to what Alasavar et al. [11] observed.

### Stability of Spray-dried Phenolic Compounds Over Time

**Fig. 2:**
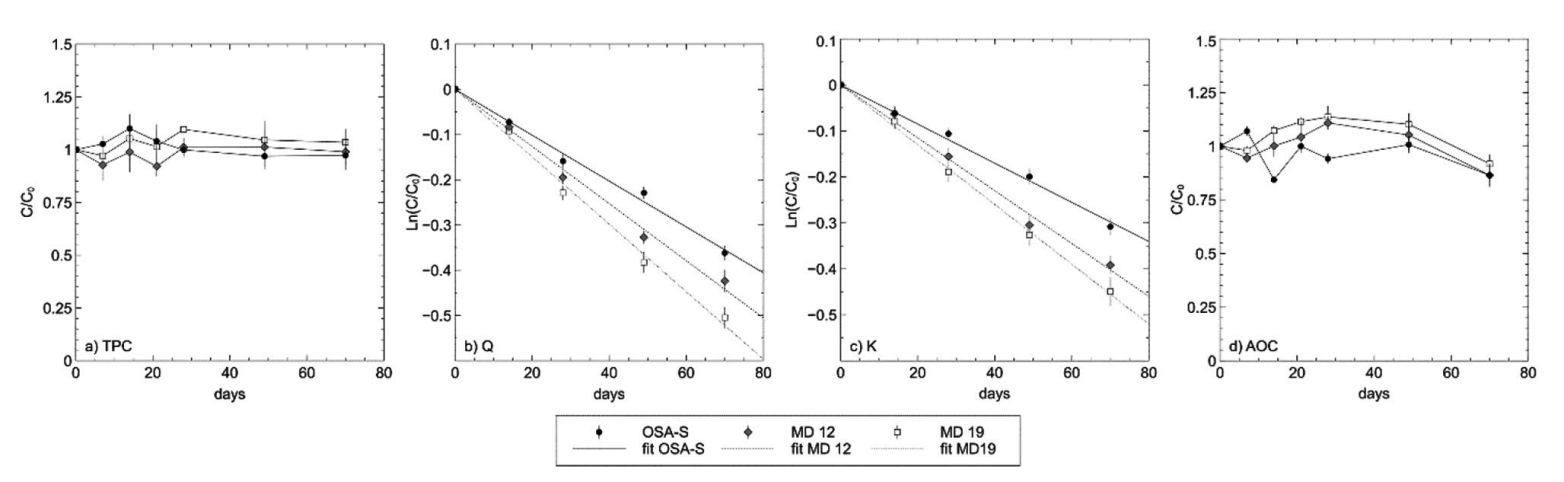
Degradation kinetics of (a) total phenolic content (TPC), (b) quercetin (Q), (c) kaempferol (K), and (d) antioxidant capacity (AOC) in the three powders during the accelerated shelf-life test. For Q and K a 1st-order fitting was proposed (*p *< 0.0001 for all regressions)

**Fig. 3:**
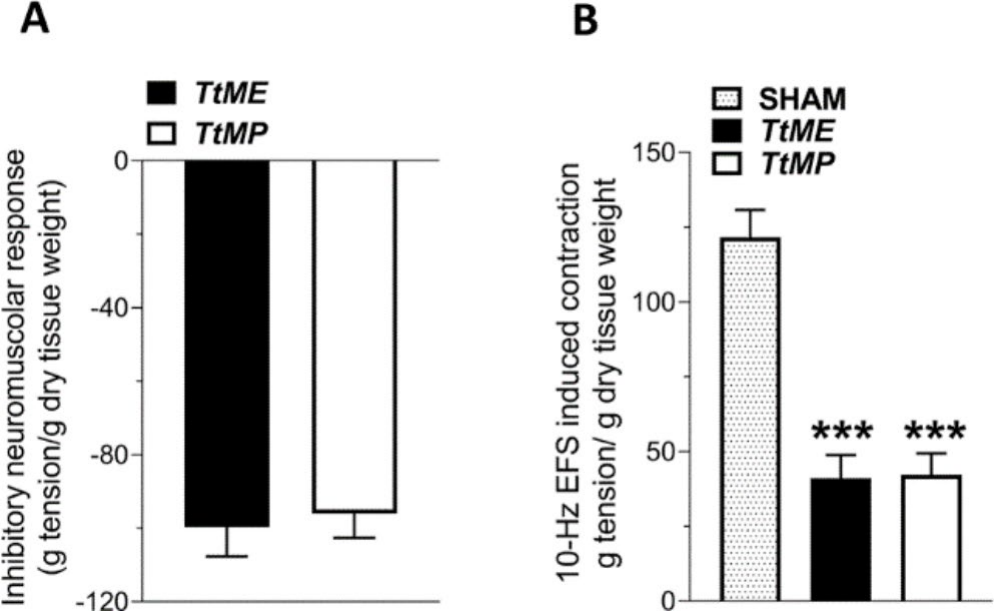
Effect of *TtMP* on small intestine contractility. (A) Ileal inhibitory neuromuscular response induced by *TtMP* or *TtME.* (B) Ileal neuromuscular excitatory response induced by 10 Hz electrical field stimulation after 15 min-incubation with *TtMP* or *TtME*. Data are reported as mean ± SE. *n* = 12/experimental group. *** *p* < 0.001; *TtMP*: *Tilia tomentosa* Moench powder; *TtME*: *Tilia tomentosa* Moench extract

Despite their potential health benefits, phenolic compounds are susceptible to degradation upon heating and UV light exposure, drastically reducing their biological activity [20]. To study the stability of the encapsulated phenols, *TtMPs* were subjected to an accelerated stability test under controlled temperature (40 °C) and humidity (75% RH) conditions in the absence of light. Figure 3 shows the storage effect on TPC, TQC, TKC, and AOC, for the three *TtMP*s. We observed that the content of flavonols, in terms of quercetin and kaempferol equivalents, decreased following 1st-order kinetics (Fig. 2b and c). This result agrees with previously published data about the thermal degradation of flavonoids [21, 22]. Kinetic parameters of Q and K are presented in Table 2 and confirm that flavonols could be better protected by using OSA-S, in accordance with the higher encapsulation efficiency of this shell material (Fig. 1). Indeed, the *t*_1/2_ of OSA-S samples was on average 30 and 51% higher than the t_1/2_ of MD 12 and MD 19 samples, respectively. The greater stability of kaempferol compared to quercetin is probably related to the number of -OH groups, *i.e*., five for quercetin and four for kaempferol, which leads to considering the latter as the less reactive, hence more stable [23].

**Table 2:** Kinetic parameters of quercetin and kaempferol degradation of TtMPs stored under accelerated conditions

		*k* (days^− 1^) x 10^− 4^	*t*_1/2_ (days) *	R^2^	p
OSA-S	Q	50.71 ± 1.47	[124.93, 146.89]	0.991	< 0.0001
	K	42.56 ± 1.04	[154.02, 176.05]	0.994	< 0.0001
MD 12	Q	63.17 ± 1.74	[101.66, 118.39]	0.992	< 0.0001
	K	57.48 ± 1.74	[111.45, 131.76]	0.991	< 0.0001
MD 19	Q	74.57 ± 1.85	[86.03, 98.81]	0.970	< 0.0001
	K	64.98 ± 0.92	[102.46, 110.82]	0.985	< 0.0001

*TtMPs*: *Tilia tomentosa* Moench powders; Q: quercetin, K: kaempferol, R^2^: coefficient of determination of the linear regression on log-transformed data. * 95% confidence intervals. *p*: goodness of regression statistics.

The behaviour of TPC (Fig. 2a) and AOC (Fig. 2d) was different in comparison to flavonols: in particular, we could not appreciate a net decrease of these values that, on the contrary, showed an increase after 15 days of storage with a final reduction (only for DPPH) in the last time intervals. One explanation for this phenomenon is that during the degradation of phenolic compounds, obtained products bearing new hydroxyl groups or characterized by structures capable of better delocalizing unpaired electrons or donating hydrogen atoms are formed, and this could affect the chemical reactions underlying the Folin-Ciocalteu and DPPH assays [24]. Consequently, the AOC lost during the thermal degradation of phenolic compounds could be compensated to different extents by the antioxidant capacity of the degradation products as already observed for microparticles [25, 26] and model flavonoids [27].

However, these data do not agree with previously published results on spray-drying particles of açai (*Euterpe oleracea* Mart.) extract [19]. The AOC of the powders obtained with MD, starch, and gum arabic showed a clear and net decrease of anthocyanins following a 1storder kinetics. Explanations for this discrepancy might lie in the different temperature and humidity conditions (35 °C x 52.3% RH) applied by the authors and in the different composition of the extract. Results similar to what Tonon et al. [19] described were observed by Lago and Norena [28] with respect to the total phenolic content on *Smallanthus sonchifolius* microparticles. The development of mathematical models could help understand the factors responsible for these discrepant results and predict the degradation kinetics of the phenolic content or the antioxidant capacity of microencapsulates [29].

### *TtMPIn Vitro* Activity on Intestinal Preparations

The activity of *TtMP* was assessed on isolated ileal segments by performing the organ bath technique. This assay allows the analysis of the influence of xenobiotics or receptor ligands or endogenous factors on intestinal contractility. Firstly, the direct effect of *TtMP* on ileal motor response was evaluated. As shown in Fig. 3a, *TtME* and *TtMP* determined a comparable inhibitory effect on ileal motor function. Moreover, ileal specimens preincubated with *TtME* or *TtMP* showed a comparable reduction of ileal contraction induced by 10 Hz EFS (electrical field stimulation)-induced contraction (Fig. 3b). Indeed, both *TtM* formulations exerted an inhibitory effect on basal ileal contractility as well as on cholinergically mediated responses evoked by EFS, that determines the release of endogenous neuronal acetylcholine, which interacts primarily with cholinergic receptors, expressed mainly on smooth muscle cells [8].

## Conclusions

Our findings prove that the spray-drying process of *TtM* flower extract through OSA-S is characterized by the best encapsulation efficiency, allowing to stabilize quercetin and kaempferol derivatives for longer times in comparison to MDs. OSA-S powder showed pharmacological effects on ileal motor functions similar to the one of *TtME*, suggesting that flavonols are not affected by the microencapsulation process and are accessible to the specific receptors. OSA-S *TtMP* thus represents a functional ingredient for the development of nutraceutical products ensuring the maintenance of the relaxation effect induced by *TtM per se*.

## Electronic supplementary material

Below is the link to the electronic supplementary material.

Supplementary Material 1

Supplementary Material 2

## Data Availability

All data generated or analysed during this study are included in this published article and its supplementary information files.

## Abbreviations

AOC: Antioxidant capacity
DE: Dextrose equivalent
K: Kaemferol
MD: Maltodextrin
OSA-S: Octenyl succinic anhydride-modified starch
Q: Quercetin
SKC: Surface kaempferol content
SPC: Surface phenolic content
SQC: Surface quercetin content
TFC: Total flavanoid content
TKC: Total kaempferol content
TPC: Total phenolic content
TQC: Total quercetin content
*TtM*: *Tilia tomentosa* Moench
*TtME*: *TtM* extract
*TtMP*: *TtM* powder
