# Supplementary material for: Spray-drying Microencapsulation of an Extract from Tilia tomentosa Moench Flowers: Physicochemical Characterization and in Vitro Intestinal Activity
Source: Plant Foods Hum Nutr. 2022 Aug 10;77(3):467–73. doi: 10.1007/s11130-022-00995-y (PMC9463327; doi:10.1007/s11130-022-00995-y)
Supplement: Supplementary file 1 — Supplementary Material 1 [file 11130_2022_995_MOESM1_ESM.docx]

*Plant Foods for Human Nutrition – Supplementary Material 1*

**Spray-drying microencapsulation of an extract from *Tilia tomentosa* Moench flowers: physicochemical characterization and *in vitro* intestinal activity**

Federica Mainente^1^, Anna Piovan^2^, Francesca Zanoni^3^, Roberto Chignola^1^, Silvia Cerantola^2^, Sofia Faggin^2^, Maria Cecilia Giron^2,4^, Raffaella Filippini^2^, Roberta Seraglia^5^, Gianni Zoccatelli^1,3§^.

^1^ Department of Biotechnology, University of Verona, Strada Le Grazie, 15 - 37134 Verona, Italy

^2^ Department of Pharmaceutical and Pharmacological Sciences, University of Padova, Via Marzolo,

5 - 35131 Padova, Italy

^3^ Sphera Encapsulation SRL, Via Alessandro Volta, 15A - 37062 Villafranca di Verona, Verona, Italy

^4^ IRCCS San Camillo Hospital, Via Alberoni, 70 - 30126 Venice, Italy

^5^ CNR-ICMATE, Corso Stati Uniti, 4 - 35127 Padova, Italy

^§^ Corresponding Author: Gianni Zoccatelli, PhD

Department of Biotechnology - University of Verona

Strada Le Grazie, 15 - CV1

37134 Verona, Italy

Tel: +39 045 8027952

Fax: +39 045 8027929

e-mail: gianni.zoccatelli@univr.it

**Materials and Methods**

**Materials**

Silver linden flower (*Tilia tomentosa* Moench, *TtM*) extract (*TtME*) was kindly supplied by the Agripharma company (Padua, Italy). Modified OSA-starch CAPSUL® was provided by Ingredion (Illinois, US). Maltodextrins from corn starch with dextrose equivalent (DE) values of 12 and 19, respectively, were from Agrana (Vienna, Austria). If not differently stated, all analytical grade chemicals and reagents used were purchased from Merck KGaA (Darmstadt, Germany).

**Moisture content and total solid quantification**

Moisture content was assessed gravimetrically following the AOAC 925.09 standard method (AOAC, 2016). All analyses were carried out in triplicate.

**Total Polyphenols Content**

The total polyphenols content (TPC) was determined by the Folin-Ciocalteu method, as described by Zanoni et al. [1] on a 96-well plate. Results were expressed as gallic acid equivalents (GAE) per liter of extract or mg GAE per g of powder. Each analysis was performed in triplicate.

**Total Flavonoids Content**

The total flavonoids content (TFC) was evaluated as described by Pekal and Pypzynska [2], using procedure no. 1 suitable for quantifying flavonols. Results were expressed as g of quercetin equivalents (QE) per liter of *TtME*. Each analysis was performed in triplicate.

**Determination of the Antioxidant Capacity (AOC)**

The DPPH free radical scavenging assay was performed according to a method previously described [3]. The results were expressed as g of Trolox equivalents (TE) per liter of *TtM* extract or as mg of TE per gram of powder. All the analyses were performed in triplicate.

**HPLC analysis**

HPLC analyses were carried out by an Agilent 1100 HPLC Series System (Agilent, Santa Clara, CA, USA) using a Gemini 5 µm C6-Phenyl column (250 X 4.6 mm) from Phenomenex (Torrance, CA, USA). Separations were conducted as described previously [4]. For quantification purposes, the samples were subjected to hydrolysis before HPLC separation [5].

The content of flavonols was expressed as quercetin (Q) and kaempferol (K) equivalents (QE and KE, respectively). Six-points calibration curves (5–100 µg/mL) were obtained using quercetin and kaempferol standard solutions (1 mg/mL) prepared in methanol. Peak areas were plotted against corresponding concentrations (R^2^= 0.999).

**Microencapsulation by spray-drying**

**Production of TtM powder (TtMP)**

The encapsulation process was performed by spray-drying, setting parameters proposed by Zanoni et al. [1] with minor modifications. Ethanol was removed from the *TtME* by a Rotavapor® R-200 (Büchi Labortechnik AG, Flawil, Switzerland). OSA-starch or maltodextrins were rehydrated directly in the extracts at a final concentration of 20% (w/v) for two h before their use. The atomization process was performed using a Mini-Spray dryer B-290 (Büchi), setting the parameters as follows: inlet temperature 170°C, airflow rate 600 L/h, and feed flow rate of 10.5 mL/min. The *TtMP* formed were collected in the cyclone vessel closed in aluminium sealed bags and stored at 4°C until use.

**Quantification of surface and total phenolic compounds and flavonols in TtMPs**

The surface and the total phenolic fractions of the powders were extracted as previously described [6] with some modifications. To extract the surface phenolic fraction, 200 mg of powder were mixed with 2 ml of a solution of ethanol and methanol (50:50 v/v). Samples were shaken for one minute and filtered through a 0.45 μm cellulose filter.

For the total phenolic fraction, 2 ml of a solution of methanol, water, and acetic acid (50:42:8 v/v/v) were added to the 200 mg of powder. The samples were shaken for 1 min and sonicated at room temperature for 20 min using a Branson 5210 Ultrasonic bath (Emerson Electric Co., St. Louis, MO, USA). Samples were centrifuged at 3.900 *g* for 10 min at room temperature, and the supernatants were filtered through a 0.45 μm cellulose filter. Total phenols and flavonols (Q and K) were quantified by the Folin-Ciocalteu method and HPLC, respectively. They were named total phenolic content (TPC), surface phenolic content (SPC), total quercetin content (TQC), surface quercetin content (SQC), total kaempferol content (TKC), and surface kaempferol content (SKC). AOC was analyzed only on the total phenolic fraction.

**Encapsulation efficiency (EE)**

The encapsulation efficiency was calculated by the following equation:

$EE\%=1-\frac{surface active}{total active}\times100$ (1)

where the surface active is SPC, SQC, or SKC, and the total active is TPC, TQC, or TKC.

**Encapsulation yield (EY)**

The encapsulation yield (EY) was calculated as a percentage of the ratio between the *TtMP* collected at the end of the process, and the amount of solid used to feed the spray-drying system initially.

The EY was expressed as:

$EY\%=\frac{g of collected powder}{g of wall material+g of total solid of the extract}\times100$ (2)

**Water activity**

The water activity (Aw) of the *TtMPs* was measured in triplicate using a Rototronic HygroPalm device (Rototronic AG, Bassersdorf, Switzerland) at 25 ± 1°C.

**Stability test (Accelerated shelf-life test)**

An accelerated shelf-life test of *TtMPs* was carried out by incubating the samples place in Petri dishes inside a thermostatic chamber (Memmert, Germany) at 40°C and 75% RH for 70 days. Samples were collected at the time points indicated in Figure 2 and used for the different experiments. The 1^st^-order reaction rate constant (k) was estimated by fitting experimental data with the equation:

ln($\frac{C_{t}}{C_{0}}$) = -*kt* (3)

from where the half-life (*t_1/2_*) was calculated according to:

*t*_1/2_ = $\frac{ln2}{k}$ (4)

where C_0_ is the initial concentration of the target molecules and C_t_ is the concentration at time *t*.

**TtMP in vitro activity on intestinal preparations**

Segments, freshly isolated from mouse ileum, were excised and placed in Krebs solution for *in vitro* contractile and relaxing responses as previously described [4, 7]. Full-thickness 1 cm distal ileum segments were mounted in 10 mL organ baths and allowed to equilibrate for 45 min in Krebs solution, oxygenated with 95%/5% of O_2_/CO_2_ and maintained at 37°C in 0.5 g-resting tension. The mechanical activity of ileum segments was recorded by isometric transducers (World Precision Instruments, Berlin, Germany) connected to a PowerLab 4/30 system (ADInstruments, Oxford, UK). After 30 min-equilibration, ileal segments were stretched passively to an initial tension of 0.1 g and brought to their optimal point of length-tension relationship using 1 μM carbachol (CCh) [8]. To assess the effect of *TtM* on basal small intestinal tension, the preparations were treated with *TtMP* or *TtME* (12 µg/mL in terms of flavonol equivalents, i.e., quercetin + kaempferol) or vehicle (SHAM). To further investigate the effect of *TtMP*, ileal tissues were initially incubated for 15 min *TtMP* or *TtME* (12 µg/mL in terms of flavonol equivalents, i.e., quercetin + kaempferol) and then exposed to electrical field stimulation (EFS, 10 Hz, 40 V) using platinum electrodes connected to an S88 stimulator (Grass Instrument) to evaluate excitatory cholinergic response. Contractile responses were expressed as gram tension/gram dry tissue weight of ileal segments [4].

**Statistical Analysis**

Statistical analyses were performed using the GraphPad Prism version 8.0.0 for Windows (GraphPad Software, San Diego, CA, USA) and the data expressed as mean ± standard error of the mean (SE). Differences between the experimental groups were assessed using unpaired Student’s t-test and, where appropriate, by one-way analysis of variance (ANOVA), followed by the post hoc Tukey test. “N” indicates the number of ileal segments.

**References**

1. Zanoni F, Primiterra M, Angeli N, Zoccatelli G (2020) Microencapsulation by spray-drying of polyphenols extracted from red chicory and red cabbage: Effects on stability and color properties. Food Chem 307:125535. <https://doi.org/10.1016/j.foodchem.2019.125535>

2. Pekal A, Pyrzynska K (2014) Evaluation of Aluminium Complexation Reaction for Flavonoid Content Assay. Food Anal Method 7(9):1776-1782. <https://doi.org/10.1007/s12161-014-9814-x>

3. Vakarelova M, Zanoni F, Lardo P, Rossin G, Mainente F, Chignola R, Menin A, Rizzi C, Zoccatelli G (2017) Production of stable food-grade microencapsulated astaxanthin by vibrating nozzle technology. Food Chem 221:289-295. <https://doi.org/10.1016/j.foodchem.2016.10.085>

4. Cerantola S, Faggin S, Annaloro G, Mainente F, Filippini R, Savarino EV, Piovan A, Zoccatelli G, Giron MC (2021) Influence of *Tilia tomentosa* Moench extract on mouse small intestine neuromuscular contractility. Nutrients 13(10):3505. <https://doi.org/10.3390/nu13103505>

5. Innocenti G, Piovan A, Filippini R, Caniato R, Cappelletti EM (1997) Quantitative recovery of furanocoumarins from *Psoralea bituminosa*. Phytochem Analysis 8(2):84-86. <https://doi.org/Doi> 10.1002/(Sici)1099-1565(199703)8:2<84::Aid-Pca336>3.3.Co;2-N

6. Robert P, Gorena T, Romero N, Sepulveda E, Chavez J, Saenz C (2010) Encapsulation of polyphenols and anthocyanins from pomegranate (*Punica granatum*) by spray drying. Int J Food Sci Tech 45(7):1386-1394. <https://doi.org/10.1111/j.1365-2621.2010.02270.x>

7. Cerantola S, Caputi V, Contarini G, Mereu M, Bertazzo A, Bosi A, Banfi D, Mantini D, Giaroni C, Giron MC (2021) Dopamine Transporter Genetic Reduction Induces Morpho-Functional Changes in the Enteric Nervous System. Biomedicines 9(5):465. <https://doi.org/https://doi.org/10.3390/biomedicines9050465>

8. Marsilio I, Caputi V, Latorre E, Cerantola S, Paquola A, Alcalde AI, Mesonero JE, O'Mahony SM, Bertazzo A, Giaroni C, Giron MC (2021) Oxidized phospholipids affect small intestine neuromuscular transmission and serotonergic pathways in juvenile mice. Neurogastroent Motil 33(4):e14036. <https://doi.org/10.1111/nmo.14036>
