# Supplementary material for: Spray-drying Microencapsulation of an Extract from Tilia tomentosa Moench Flowers: Physicochemical Characterization and in Vitro Intestinal Activity
Source: Plant Foods Hum Nutr. 2022 Aug 10;77(3):467–73. doi: 10.1007/s11130-022-00995-y (PMC9463327; doi:10.1007/s11130-022-00995-y)
Supplement: Supplementary file 2 — Supplementary Material 2 [file 11130_2022_995_MOESM2_ESM.docx]

*Plant Foods for Human Nutrition – Supplementary Material 2*

**Spray-drying microencapsulation of an extract from *Tilia tomentosa* Moench flowers: physicochemical characterization and *in vitro* intestinal activity**

Federica Mainente^1^, Anna Piovan^2^, Francesca Zanoni^3^, Roberto Chignola^1^, Silvia Cerantola^2^, Sofia Faggin^2^, Maria Cecilia Giron^2,4^, Raffaella Filippini^2^, Roberta Seraglia^5^, Gianni Zoccatelli^1,3§^.

^1^ Department of Biotechnology, University of Verona, Strada Le Grazie, 15 - 37134 Verona, Italy

^2^ Department of Pharmaceutical and Pharmacological Sciences, University of Padova, Via Marzolo,

5 - 35131 Padova, Italy

^3^ Sphera Encapsulation SRL, Via Alessandro Volta, 15A - 37062 Villafranca di Verona, Verona, Italy

^4^ IRCCS San Camillo Hospital, Via Alberoni, 70 - 30126 Venice, Italy

^5^ CNR-ICMATE, Corso Stati Uniti, 4 - 35127 Padova, Italy

^§^ Corresponding Author: Gianni Zoccatelli, PhD

Department of Biotechnology - University of Verona

Strada Le Grazie, 15 - CV1

37134 Verona, Italy

Tel: +39 045 8027952

Fax: +39 045 8027929

e-mail: gianni.zoccatelli@univr.it

| **Table S1. Chemical characteristics of *Tilia tomentosa Moench extract*** | | | | | | | | | | | |
| --- | --- | --- | --- | --- | --- | --- | --- | --- | --- | --- | --- |
| **TPC**  **(mg GAE/mL)** | | **TFC**  **(mg QE/mL)** | | **AOC (DPPH)**  **(mg TE/mL)** | | **Q**  **(μg QE/mL)*** | | **K**  **(μg KE/mL)*** | | **Solid content**  **(g/L)** | |
| mean | SE | mean | SE | mean | SE | mean | SE | mean | SE | mean | SE |
| 11.22 | 0.68 | 0.72 | 0.03 | 19.70 | 0.92 | 399* | 2.8 | 252* | 2.1 | 76.6 | 2.1 |
| TPC: total phenolic content; TFC: total flavonoid content AOC: antioxidant capacity; Q: quercetin content; K: kaempferol content. Values are expressed as mean ± standard error (SE) of three independent measurements. *These data were obtained in a previous study [1]. | | | | | | | | | | | |

Q*


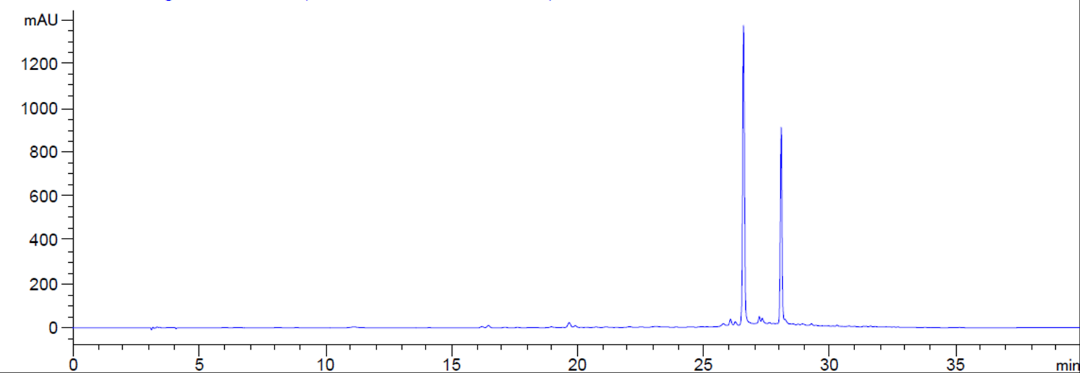

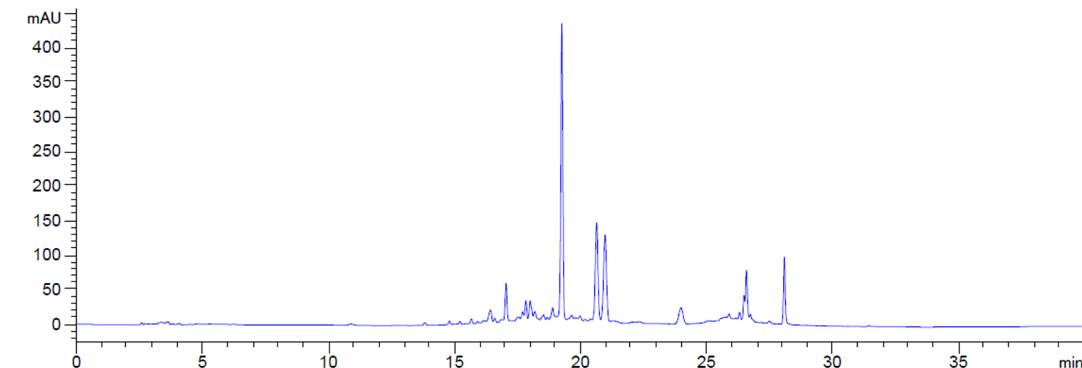


K

Q

**b**

**a**

Q*

K

K*

Q

K*

K*

Figure S1. HPLC chromatograms of *Tilia tomentosa* Moench extract (*TtME*) recorded at 365 nm. The chromatogram (**a**) shows the *TtME* profile; the analysis of UV spectra led to the identification of the peaks at 17.0, 20.9, 24.0 min as kaempferol derivatives (K*), peaks at 19.2, 21.2 min as quercetin derivatives (Q*), and peaks at 26.7 and 28.1 min as quercetin (Q) and kaempferol (K) aglycones. The chromatogram (**b**) shows the profile of the hydrolyzed *TtME* in which only the peaks of quercetin (Q) and kaempferol (K) aglycones are visible at 26.7 and 28.1 min, respectively.


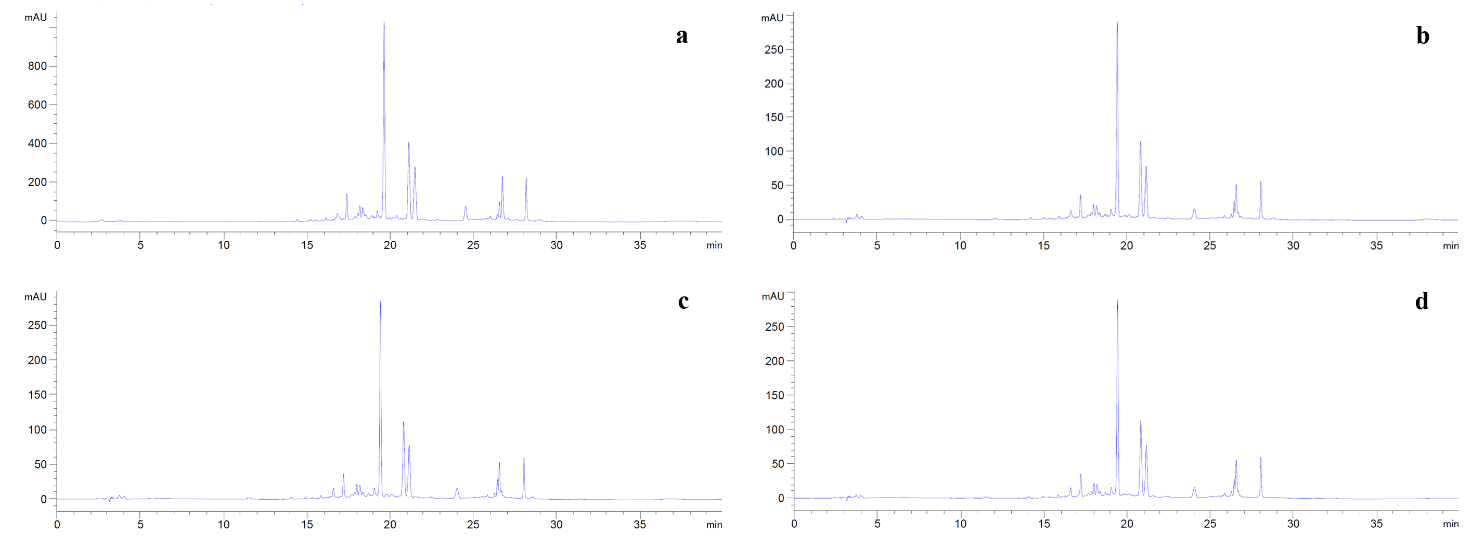


Figure S2. HPLC chromatograms recorded at 365 nm of *Tilia tomentosa* Moench extract (a) and *Tilia tomentosa* Moench powders obtained using OSA-S (b), MD12 (c), and MD19 (d).

**References**

1. Cerantola S, Faggin S, Annaloro G, Mainente F, Filippini R, Savarino EV, Piovan A, Zoccatelli G, Giron MC (2021) Influence of *Tilia tomentosa* Moench extract on mouse small intestine neuromuscular contractility. Nutrients 13(10):3505. <https://doi.org/10.3390/nu13103505>
